# Anterior Column Reconstruction of Destructive Vertebral Osteomyelitis at the Thoracolumbar Spine with an Expandable Vertebral Body Replacement Implant: A Retrospective, Monocentric Radiological Cohort Analysis of 24 Cases

**DOI:** 10.3390/jcm13010296

**Published:** 2024-01-04

**Authors:** Lisa Klute, Marie Esser, Leopold Henssler, Moritz Riedl, Melanie Schindler, Markus Rupp, Volker Alt, Maximilian Kerschbaum, Siegmund Lang

**Affiliations:** Clinic of Trauma Surgery, University Medical Center Regensburg, Franz-Josef-Strauss-Allee 11, 93053 Regensburg, Germany

**Keywords:** expandable vertebral body replacement, vertebral osteomyelitis, bony fusion rate, anterior column reconstruction, spondylodiscitis

## Abstract

Background: Vertebral osteomyelitis (VO) often necessitates surgical intervention due to bone loss-induced spinal instability. Anterior column reconstruction, utilizing expandable vertebral body replacement (VBR) implants, is a recognized approach to restore stability and prevent neurological compromise. Despite various techniques, clinical evidence regarding the safety and efficacy of these implants in VO remains limited. Methods: A retrospective cohort analysis, spanning 2000 to 2020, was conducted on 24 destructive VO cases at a Level 1 orthopedic trauma center. Diagnosis relied on clinical, radiological, and microbiological criteria. Patient demographics, clinical presentation, surgical interventions, and radiological outcomes were assessed. Results: The study included 24 patients (62.5% male; mean age 65.6 ± 35.0 years), with 58% having healthcare-associated infections (HAVO). The mean radiological follow-up was 137.2 ± 161.7 weeks. Surgical intervention significantly improved the bi-segmental kyphotic endplate angle (BKA) postoperatively (mean −1.4° ± 13.6°). However, a noticeable loss of correction was observed over time. The study reported a mortality rate of 1/24. Conclusions: Anterior column reconstruction using expandable VBR effectively improved local spinal alignment in destructive VO. However, the study underscores the necessity for prolonged follow-up and continuous research to refine surgical techniques and postoperative care. Addressing long-term complications and refining surgical approaches will be pivotal as the field progresses.

## 1. Introduction

Vertebral osteomyelitis (VO) is the most common manifestation of osteomyelitis in the adult population with an increasing incidence rate [1,2]. A feared complication is spinal instability due to progressive bone loss of the infected vertebral bodies (VB) [3]. However, VO requires complete surgical debridement only in chosen, severe cases [4]. In most other cases, conservative treatment, or a limited surgical regime is sufficient. In cases in which surgery is indicated, it has been suggested to significantly reduce pain, enhance neurologic function, and result in a high percentage of patients going back to their previous functional/work status [5]. In these cases, segmental stabilization with dorsal instrumentation and the option of interbody fusion, combined with systemic antibiotic therapy, usually is the therapy of choice [6,7]. Indications for surgical treatment of pyogenic spondylodiscitis are sepsis, an epidural abscess, neurological deficits/complications, and instabilities/deformities in the affected motion segment, which are also included in current classification systems [8]. Preservation of vertebral body integrity, development of spinal deformities, refractory back pain, inadequate patient compliance, and failure of conservative therapy are considered relative surgical indications. Segmental kyphosis >15°, vertebral body loss >50%, and/or translation >5 mm are considered instability criteria [9,10]. Combined posterior–anterior surgery is considered in cases of large anterior defects. It has been demonstrated that a better reconstruction of the sagittal profile could be achieved with posterior–anterior stabilization in comparison to posterior only constructs [11]. The indication for the posterior–anterior stabilization [12] mainly depends on the clinical course, radiological parameters for segmental instability, and on the experience of the treating surgeons [13,14]. An autologous iliac bone crest can be used to restore the ventral load-bearing column. This has the disadvantage of donor site morbidity and the risk of subsequent graft failure due to collapse of the bone chip. Alternatively, cages filled with autologous bone or, in the case of larger defects, (expandable) vertebral body replacement implants can be used. The titanium cage is considered the gold standard, although polyetheretherketone (PEEK) cages show comparable results in the medium term [15]. Expandable vertebral body replacement (VBR) implants have been shown to be an effective alternative for bone blocks and cages [16,17,18,19]. Data on restoring the bi-segmental kyphotic endplate angle (BKA) in VO patients are scarce, despite the fact that it has been demonstrated that they are effective in supplying primary stability. It is noteworthy that reports have been made of cage subsidence and loss of ventral support over time [20,21]. The ensuing kyphotic malalignment may also cause subsequent neurological signs and diseases, impairing spinal function [22,23].

This study aimed to examine the safety and radiological outcome of posterior–anterior treatment with anterior column reconstruction of destructive vertebral osteomyelitis at the thoracolumbar spine with an expandable VBR (ObeliscTM, Ulrich Medical, Ulm, Germany).

## 2. Materials and Methods

This retrospective study conducted at a Level 1 orthopedic trauma center in Germany focused on patients with spondylodiscitis who underwent vertebral body replacement (VBR) between 1 January 2000 and 31 December 2020. The evaluation considered three time points: pre-operation, post-operation, and the final follow-up, ensuring a minimum follow-up period of 6 weeks.

### 2.1. Patient Selection and Characterization

Patients eligible for this study were those aged 18 years or older diagnosed with vertebral osteomyelitis (VO) per ICD-10 codes: M46.2 (osteomyelitis of vertebra), M46.3 (infection of the intervertebral disc, pyogenic), M46.4 (discitis, unspecified), and M46.5 (other infective spondylopathies). The cases were meticulously screened, and the diagnosis was confirmed by compatible clinical features, radiological evidence in CT and/or MRI, and microbiologic demonstration of bacterial pathogens.

The study differentiated between healthcare-associated vertebral osteomyelitis (HAVO) and community-acquired vertebral osteomyelitis (CAVO) [24,25]. HAVO was identified if symptoms developed a month after hospitalization without prior evidence of VO, or if there was a hospital admission or outpatient diagnostic or therapeutic manipulation six months before symptom onset. If none of these criteria were met, VO cases were classified as CAVO.

The inclusion criterion was patients with vertebral osteomyelitis in the thoracolumbar spine who were treated with a VBR implant and had at least two radiological follow-ups after surgery, with the latter one after a minimum follow-up period of 6 weeks. Exclusion criteria were patients under 18, those with non-operative treatment or surgical treatment other than an expandable VBR, and those with incomplete radiological follow-up. Given the retrospective nature of the data-set, VO patients with heterogeneous infection courses were indicated for ventral column reconstruction. In general, we consider vertebral body replacement for defects encompassing 50% or more of the vertebral body, progressive osteolysis, persistent symptoms despite dorsal instrumentation and antibiotic therapy, and cases with severe local sagittal deformity. The indication primarily depended on the individual patient’s situation.

### 2.2. Data Collection and Ethics

Data were retrospectively collected, focusing on patient demographics, injury mechanism, neurological status, treatment details, and microbiological details on the causative pathogens and treatments. Septic patients admitted to the Intensive Care Unit were classified according to the Sequential Organ Failure Assessment score. The study adhered to the Declaration of Helsinki and was approved by the University of Regensburg’s ethics committee (Number: 12-218_2-101 09/2021).

### 2.3. Radiological Assessment

**Figure 1:**
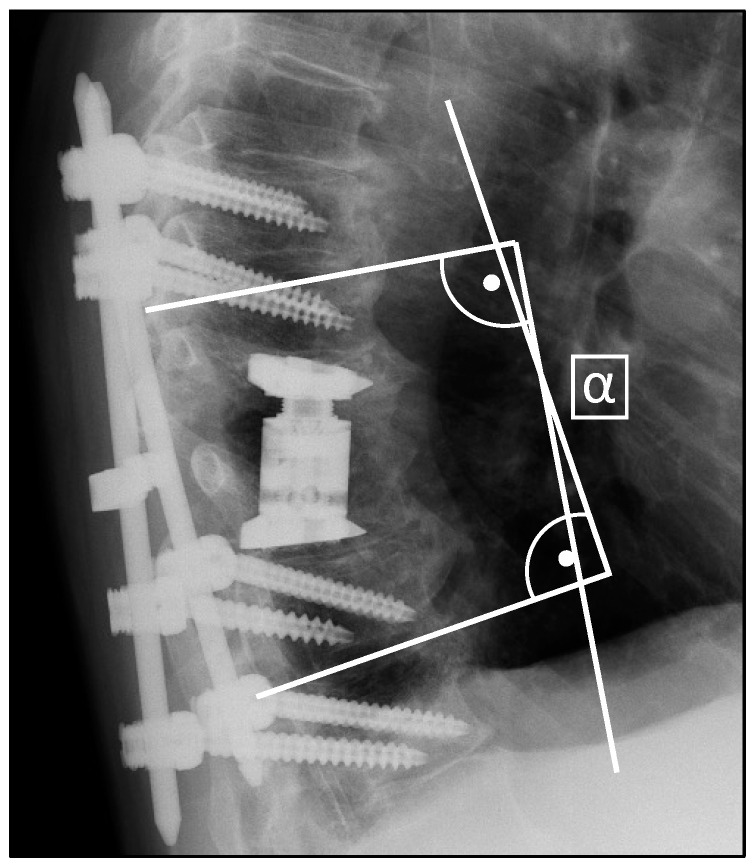
Measurement of the bi-segmental kyphotic endplate angle (BKA) in medio-lateral X-rays.

Radiological assessment involved pre- and postoperative CT scans and X-rays for surgical planning and implant verification, with subsequent X-rays conducted at least 6 weeks post-surgery. The evaluation utilized the bi-segmental kyphotic endplate angle (BKA), visualized in Figure 1, to measure medio-lateral X-rays. In the assessment, BKA values below zero denote kyphosis, while values above zero denote lordosis. In a subset analysis, we separately evaluated the BKA at the thoracic spine (T1-T10), thoracolumbar transition (T11-L2), and the lumbar spine (L3-L5). Fusion at the final follow-up was assessed using the Bridwell [26] classification system. Briefly, the evaluation of fusion rates was conducted via lateral X-ray examination, where the absence of radiolucency, lack of bone sclerosis, and presence of bridging trabecular bone within the fusion area were assessed. Additionally, the observation of screw loosening or implant displacement indicated the presumption of insufficient fusion.

### 2.4. Statistical Analysis

Statistical analysis was conducted using SPSS software, version 28. Tests, including Mann–Whitney U, Kruskal–Wallis, and independent *t*-tests, were performed as appropriate. The associations between implant specifications and the loss of correction were assessed using Pearson correlation analysis. *p*-values < 0.05 were deemed statistically significant. Continuous variables are presented as mean ± standard deviation, and categorical data as frequencies.

## 3. Results

**Figure 2:**
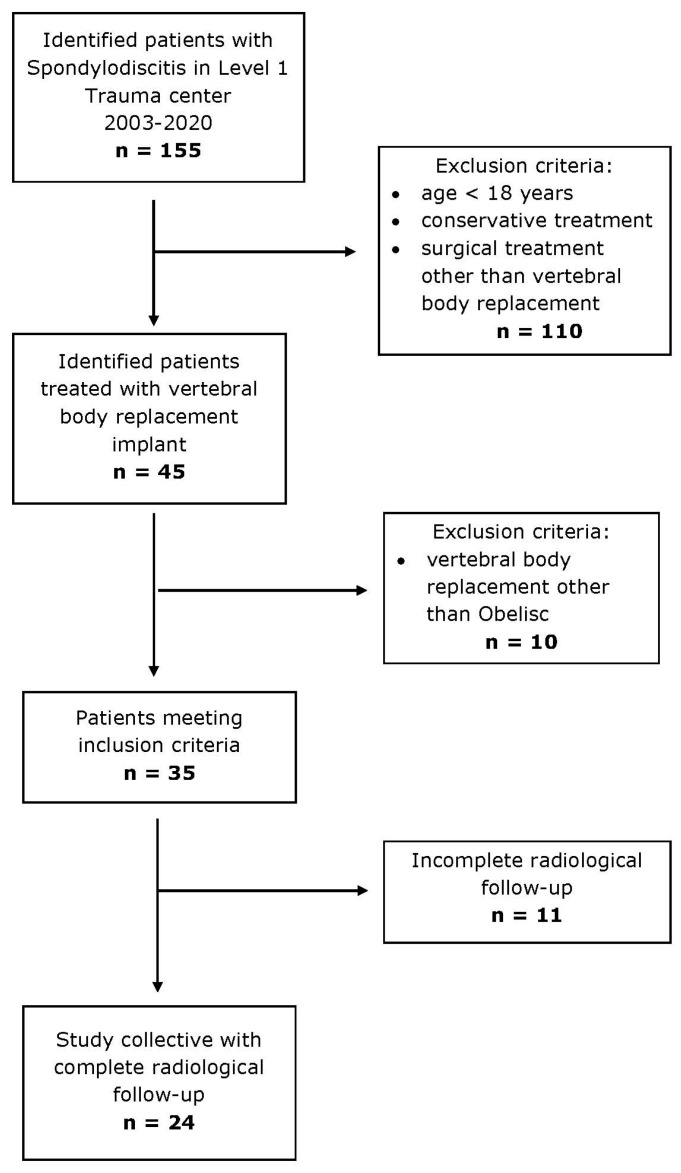
Inclusion and exclusion criteria of study cohort.

The study included 24 patients (Figure 2), with a male predominance (62.5%) and a mean age of 65.6 ± 35.0 years. The average BMI was 29.5 ± 6.3. Symptoms had been present for 71.0 ± 46.3 days on average before hospitalization. Healthcare-associated infections (HAVO) were identified in 58% of cases (Table 1), with 54.2% potentially being iatrogenic. The mean hospital stay was 33.2 ± 22.3 days, and 75.0% (18/24) of patients required ICU (Intensive Care Unit) admission, with an average ICU (Intensive Care Unit) stay of 3.3 ± 3.4 days. A third of the patients (33.3%) developed sepsis during their hospitalization, according to the reviewed diagnoses in the patient charts. Initial CRP levels upon admission were high at 149.3 ± 11.2 mg/L, decreasing to a mean of 83.6 ± 9.7 mg/L by the end of the hospitalization. Leukocyte counts increased slightly from an initial mean of 9.4 ± 3.5 × 10^9^/L to a final mean of 10.0 ± 7.5 × 10^9^/L.

Six (25.0%) patients had a paravertebral abscess, eight (33.3%) had a psoas abscess, and three (12.5%) had an epidural abscess. Neurological complications were also present: eight (33.3%) patients had paresis, two (8.3%) had hyposensitivity, and two (8.3%) had paresthesia. Six (25.0%) patients experienced a spinal cord injury. Additionally, 19 patients (79.2%) reported back pain.

An acute fulminant septic course was observed in eight cases (33.4%). With *Staphylococcus aureus* being the most isolated pathogen (*n* = 10, 41.7%), *Streptococcus* species (*n* = 1, 4.2%) and *Enterococcus* (*n* = 1, 4.2%) were detected in patients’ microbiological samples. In 12 cases, no pathogen could be isolated. The most frequent comorbidity was diabetes mellitus (*n* = 10, 41.7%), followed by congestive heart failure (*n* = 7, 29.2%). Perioperative abscess formation occurred in 15 cases (62.5%), with percutaneous drainage performed in 11 cases. All patients received empirical broad-spectrum antibiotic therapy, which was subsequently adjusted according to antibiotic susceptibility testing in cases where pathogens were identified. Nine patients were treated with antibiotic monotherapy, while the remaining patients received combination antibiotic regimens, including the addition of rifampicin in five cases, among other variations. The mean duration of antibiotic therapy was 62.4 ± 28 days.

Among the cohort, 22 (91.6%) cases underwent dorsal instrumentation. The reconstruction of the anterior column and implantation of the VBR was conducted using a thoracoscopic approach in *n* = 11 cases (45.8%) and a lumbotomy in *n* = 4 (16.7%) cases, whereas in *n* = 9 (37.5%) cases an isolated dorsal approach was used. In 20 cases (83.3%), implants with 0° angulation base- and endplates were used, while in two cases (8.3%) 5° and 10° angulations were utilized, respectively. A total of seven different VBR sizes were utilized, ranging from 17–23 mm to 40–62 mm.

A total of 17 patients underwent a one-staged procedure, and in seven cases a two-staged procedure was performed. The mean surgical duration for the reconstruction of the anterior column was calculated at 155.5 ± 65 min. Complications were observed in six patients (25.0%), encompassing a dislocation of the VBR implant in one case (4.2%), material irritation in three cases (12.5%), postoperative hematoma in one case (4.2%), and screw dislocation in one case (4.2%). Among these cases, five (20.8%) required subsequent revision surgeries to address the identified complications. In the study population, material irritation manifested as localized discomfort and pain at the site of the implanted dorsal instrumentation material, while VBR dislocation, observed radiographically during follow-up, did not exhibit any clinical symptoms. However, it necessitated implant removal due to a high risk of potential further damage. The time to failure, defined as the need for revision surgery, was recorded at an average of 280.5 ± 386.8 days (as shown in Table 2). The in-hospital mortality rate was observed to be 4.2% (*n* = 1). Recurrent infection related to the VBR was not recorded during the follow-up period.

### Radiological Outcome

**Figure 3:**
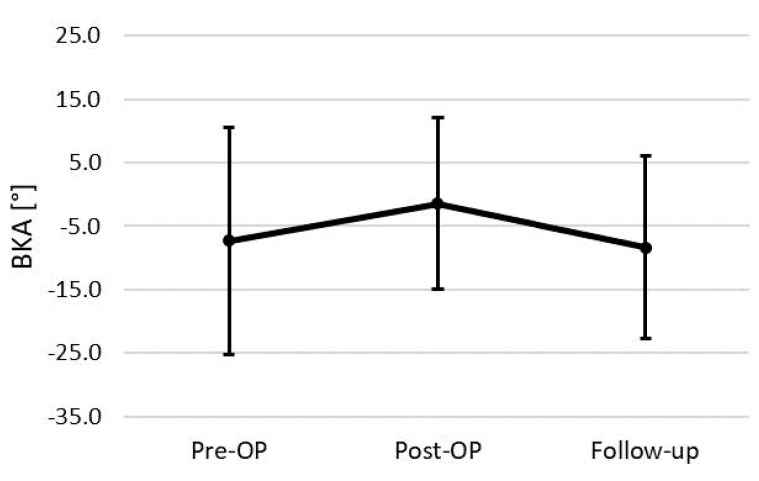
BKA changes of the total cohort (*n* = 24).

The mean radiological follow-up was after 137.2 ± 161.7 weeks with a minimum follow-up of 6 weeks. Examining the entire cohort initially, we found a mean preoperative BKA of −7.3° (±17.9°). Post-surgery, a significant correction in the BKA was observed, demonstrated by a postoperative mean of −1.4° (±13.6°; *p* = 0.023). At the follow-up, we observed a decrease of the BKA to a mean of −8.3° (±14.4°), indicating a significant loss of surgical correction over time by 8.7 ± 7.7° (*p* < 0.000, Figure 3).

**Figure 4:**
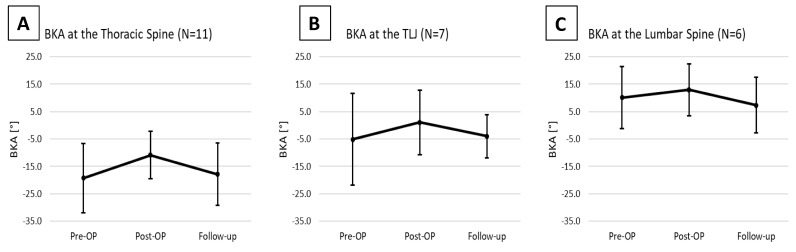
Changes in the BKA in the Thoracic Spine (**A**), the Thoracolumbar junction (**B**), and the Lumbar Spine (**C**).

For the thoracic spine subset of patients, the preoperative mean BKA was −19.3 ± 12.7°, showing a more pronounced kyphotic deformity than the overall cohort. Post-surgery, a correction was noted with a postoperative mean BKA of −10.8 ± 8.6° (*p* = 0.123). By the time of follow-up, the mean BKA had decreased to −17.8 ± 11.4°, reflecting a significant loss of correction by 8.3 ± 7.1° (*p* = 0.021; Figure 4A).

At the thoracolumbar junction the preoperative mean BKA was −5.1 ± 16.7°. Postoperative measurements indicated a corrected mean of 1.1 ± 11.7°, yet the change did not reach statistical significance (*p* = 0.245). By the time of follow-up, the mean BKA was −4.0 ± 7.8°, implying loss of correction by 11.1 ± 10.1° (*p* = 0.067, Figure 4B).

At the lumbar spine, the preoperative mean BKA was 10.1° ± 11.2°. Postoperatively, there was an observed correction to a postoperative mean of 12.9 ± 9.4°, showing a correction by 4.1 ± 3.4° (*p* = 0.210). At follow-up, the mean BKA decreased to 7.4 ± 10.2°, demonstrating a loss of correction by 6.8 ± 6.1° (*p* = 0.109; Figure 4C).

We did not detect a correlation between the VBR size and the amount of loss of correction (r = 0.178; *p* = 0.400). There was a medium positive correlation between the used VBR base-and endplate angulation and the loss of correction at the final follow-up (r = 0.513; *p* < 0.05). A subsidence rate of 5/24 was documented in patients but did not exhibit a significant correlation with changes in the BKA.

In 18 of 24 patients, we were able to assess the bony consolidation progress using the Bridwell classification [26]. Among those 18 patients, we observed varying progress in fusion at the follow-up period. Specifically, 4.2% of the patients were classified as Bridwell stage 1, 58.4% as Bridwell stage 2, 8.4% as Bridwell stage 3 and 4.2% as stage 4 (as shown in Table 2).

## 4. Discussion

The present study provides a comprehensive analysis of the surgical treatment and vertebral body replacement in patients with VO, focusing on the thoracolumbar spine. Our findings suggest that surgical intervention, specifically posterior–anterior treatment with anterior column reconstruction using an expandable VBR (ObeliscTM, Ulrich Medical, Ulm, Germany), can significantly improve the bi-segmental kyphotic endplate angle (BKA) postoperatively. However, a significant loss of this surgical correction was observed over time, indicating the potential for long-term complications, cage subsidence and the need for further research and improvement in surgical techniques and postoperative care.

Our findings align with previous studies that have highlighted the effectiveness of surgical intervention in spondylodiscitis cases. For instance, a study by Kehrer et al. reported that surgical intervention could significantly reduce pain, enhance neurological function, and enable a high percentage of patients to return to their previous functional or work status [27]. Similarly, our study found that surgical intervention could significantly improve the BKA postoperatively, suggesting an improvement in spinal alignment and potentially reducing pain and enhancing neurological function. However, this study did not include a detailed clinical follow-up. It has been recently demonstrated that spondylodiscitis patients show acceptable but impaired patient-reported outcome measurements, regardless the therapy strategy [15]. A recent study by Neuhoff et al. demonstrated a sagittal correction of 10° (range 0–54°) in a cohort of 100 patients with spinal infections treated via vertebral body replacements. This aligns with our study’s results, which indicated an approximate average 6° correction in the BKA [28].

Our study also revealed a significant loss of surgical correction over time, as evidenced by the decrease in the BKA at follow-up. This finding is consistent with previous reports of cage subsidence and loss of ventral support over time in thoracolumbar vertebral body replacement [19,29]. The ensuing kyphotic malalignment may also cause subsequent neurological impairment, limiting spinal function [22,23]. This suggests that while surgical intervention can provide immediate relief and improvement, long-term complications may arise.

In the cohort studied by Neuhoff et al., 31 patients were followed up for more than one year. They found that the main causes for revision surgery were wound healing disorders (12%) and implant failure (11%), with specific complications including posterior pedicle screw loosening (8%) and anterior cage subsidence (3%) [28]. Aseptic mechanical complications were more common in longer pedicle-screw constructs, occurring significantly less in shorter constructs (0–4 levels). In comparison, 6/24 patients in our study experienced complications, including VBR implant dislocation (*n* = 1), material irritation (*n* = 3), postoperative hematoma (*n* = 1), and screw dislocation (*n* = 1), leading to revision surgeries in five cases. The average time until the unplanned revision surgery was 280.5 days. A subsidence rate of 5/24 was documented in patients but did not exhibit a significant correlation with changes in the BKA. A subsidence rate of 5 out of 24 was noted, but this did not show a significant association with alterations in the BKA during the overall follow-up period. The study’s findings underscore the complexity of managing VO, particularly within the thoracolumbar region, and depending on the complexity of the surgical intervention, due to the intricate interplay of anatomical, biomechanical, and infectious factors.

The choice of surgical intervention for anterior column reconstruction using an expandable VBR represents a strategic approach to address the multifaceted challenges posed by VO. However, it is important to acknowledge that surgical management in this context demands meticulous patient selection, thorough preoperative planning, and a nuanced assessment of risk factors to ensure optimal outcomes [2].

Furthermore, the noted decrease in the BKA over time highlights the ongoing biomechanical challenges associated with vertebral body replacement. Darwich et al. showed overall low complication rates and a good functional outcome within their cohort treated with VBR, but also that significant height gain was associated with higher complication rates [30]. While immediate improvements in spinal alignment are evident, the long-term biomechanical implications require continuous scrutiny. The interplay between surgical correction, fusion, and load distribution underscores the importance of biomechanical studies that provide insights into the longevity and sustainability of surgical outcomes.

Additionally, a reported mortality rate of 4.2% and septic complications in one third of the cohort indicate the potential severity of VO and its associated challenges. This underlines the importance of early diagnosis, timely intervention, and comprehensive management to mitigate adverse outcomes [2,31].

In analyzing our results, the study revealed a statistically significant improvement in the bi-segmental kyphotic endplate angle (BKA) postoperatively across the entire cohort, emphasizing the effectiveness of posterior–anterior treatment with anterior column reconstruction using an expandable VBR. However, the observed long-term loss of surgical correction underscores the need for continuous scrutiny and further research to address potential complications such as cage subsidence, contributing to the ongoing biomechanical challenges associated with vertebral body replacement [32,33].

### Limitations

An important limitation of our study is the unavailability of longstanding X-rays for all the cases to assess global spinal alignment. In our study, follow-up X-rays were conducted with the patient standing upright. However, we did not apply X-rays of the whole spine because global deformity correction was not the main surgical goal for the mostly severely ill patients, but rather local reconstruction and stabilization. It is worth noting that comparison between the pre- and postoperative kyphotic angle might partially be influenced by the difference of patient positioning [34]. While the preoperative CT was conducted with the patient in supine position, the postoperative follow-up X-rays were taken with the patient standing upright. Additionally, beyond these considerations, 11 patients were excluded from the analysis because of incomplete radiological follow-up. The reported follow-up time of 137 ± 161.7 weeks exhibits a notably uneven distribution. This asymmetry in follow-up duration is a relevant consideration that may have influenced the interpretation of the radiographic results. Another limitation is that our retrospective design restricts our study’s capability to thoroughly analyze the multifaceted factors, including heterogeneous infection courses in VO patients, that influenced the decision for patients to undergo either one- or two-staged procedures. Generally, the two-staged procedure is selected for patients with multimorbidity, as it allows for a more cautious surgical approach, minimizing operative time and blood loss. Furthermore, it is worth emphasizing that a more comprehensive understanding of the reasons behind the loss of correction is desirable, for example through a comparison with another patient group. This could be achieved through the implementation of a prospectively designed study, which might also include an evaluation of the height of the VBR in correlation to postoperative outcomes. Such an approach could provide valuable insights into optimizing patient care and surgical strategies in the future. Additionally, the evaluation of the role of osteoporosis, with VBR combined allo- or autografts as well as local antibiotics, should be taken into consideration.

In light of the retrospective design and limited sample size acknowledged in our study, it is imperative to recognize that our findings offer a valuable mid-term analysis of surgical interventions for vertebral osteomyelitis in the thoracolumbar spine. While the study’s limitations necessitate careful consideration, the observed biomechanical challenges and long-term complications underscore the importance of ongoing research and refinement in surgical techniques and postoperative care to optimize patient outcomes in this complex clinical scenario. Also, due to the retrospective design of our study, a notable limitation is the absence of an assessment of functional outcomes or other patient-associated symptoms, which could have provided a more comprehensive understanding of the clinical impact of the procedures. The potential influence of patient positioning on the comparison between pre- and postoperative kyphotic angles emphasizes the importance of standardized imaging protocols in future studies, with a recommendation for consistent positioning, such as standing lateral radiographs, to ensure more accurate and reliable measurements.

While the study has certain limitations inherent to its retrospective design and small sample size, its comprehensive analysis, clinical relevance, and focus on mid-term considerations contribute valuable insights into the outcomes of surgical interventions for VO. The findings provide a foundation for further research and optimization of treatment strategies to improve patient outcomes in this challenging clinical scenario.

## 5. Conclusions

In conclusion, the study’s radiological outcomes highlight the promising potential of surgical interventions in the management of spondylodiscitis, specifically within the thoracolumbar spine. The immediate benefits in spinal alignment improvement need to be balanced with a keen awareness of potential long-term complications and the imperative for ongoing research to refine surgical techniques and postoperative care. As the field advances, collaborative efforts that integrate biomechanical, clinical, and microbiological insights will be essential in optimizing treatment strategies and enhancing patient outcomes in this complex clinical scenario.

## Figures and Tables

**Table 1:** Baseline statistics of study cohort.

		Percentage [n]
*n*		100.0% [24]
age [years]		65.9 ± 11.9
sex		♂ = 62.5% [15]
		♀ = 37.5% [9]
BMI		29.5 ± 6.3
HAVO/CAVO [n]		HAVO = 58.4% [14]
		CAVO = 41.7% [10]
hospital stay duration [days]		33.2 ± 22.3
intensive care unit stay		75.0% [18]
intensive care unit stay duration [days]		3.3 ± 3.4
duration of symptoms [days]		71 ± 46.3
mortality (in-hospital)		4.2% [1]
localisation	thoracic spine	45.9% [11]
	thoracolumbal junction	29.2% [7]
	lumbar spine	25.0% [6]
abscess	no abscess	37.5% [9]
	total	62.5% [15]
	paravertebral	25.0% [6]
	psoas	33.4% [8]
	epidural	12.5% [3]

**Table 2:** Complications and revisions after surgical implantation of VBR.

		Percentage [n]
n		100.0% [24]
revision surgery		20.8% [5]
time to failure [days]		280.5 ± 386.8
consolidation		75.0% [18]
Bridwell classification	I	4.2% [1]
	II	58.4% [14]
	III	8.4% [2]
	IV	4.2% [1]
complications	no complications	75.0% [18]
	total	25.0% [6]
	VBR dislocation	4.2% [1]
	material irritation	12.5% [3]
	bleeding	4.2% [1]
	screw dislocation	4.2% [1]

## Data Availability

The data presented in this study are available on request from the corresponding author. The data are not publicly available due to privacy.
